# Case Report: Pediatric Hepatic Rhabdomyosarcoma With Maximum Lifetime

**DOI:** 10.3389/fmed.2022.858219

**Published:** 2022-04-15

**Authors:** Xu Li, Xiang Li, Dian-Fei Yang, Miao Li, Hong-Qin Xu, Shuang Zheng, Pu-Jun Gao

**Affiliations:** ^1^Department of Hepatology, The First Hospital of Jilin University, Changchun, China; ^2^Department of Pediatric Oncology, The First Hospital of Jilin University, Changchun, China; ^3^Department of Pathology, The First Hospital of Jilin University, Changchun, China; ^4^Department of Radiology, The First Hospital of Jilin University, Changchun, China

**Keywords:** rhabdomyosarcoma, embryonal, hepatic, liver, diagnosis

## Abstract

Primary intrahepatic rhabdomyosarcoma is an extremely rare malignant tumor. Here, we describe a case of embryonal rhabdomyosarcoma of the liver in a 7-year-old boy without any symptoms. Serologically, the patient showed abnormal levels of serum tumor markers and liver function. Imaging revealed a large mass in the left lobe of the liver and no lesions elsewhere. At first, the patient was misdiagnosed by percutaneous liver biopsy as having clear cell sarcoma. However, the final diagnosis was established to be hepatic embryonal rhabdomyosarcoma based on postoperative histopathology and typical immunohistochemical staining, which was positive for desmin and myogenin. For treatment, the patient received two cycles of preoperative chemotherapy, prophylactic radiotherapy, and 13 cycles of combined postoperative chemotherapy. Routine follow-ups after all treatment conducted by imaging examinations showed no sign of recurrence or metastasis over 13 months, and the patient survives more than 38 months since initial diagnosis. To our knowledge, this patient is the first with hepatic rhabdomyosarcoma to receive neoadjuvant chemotherapy (preoperative chemotherapy) combined with relative comprehensive treatment and achieve a favorable result.

## Background

Rhabdomyosarcoma is the most common soft-tissue sarcoma in children and adolescents, accounting for 3% of all pediatric tumors (1). However, primary intrahepatic rhabdomyosarcoma is extremely rare. Since 1956, a total of 20 cases of hepatic rhabdomyosarcoma have been reported (2–14), and only two cases in China were published. Rhabdomyosarcoma rarely affects the liver as a primary pediatric malignancy and has poor prognosis, with a mean survival time of 9.25 months (2–14). Thus, the clinical diagnosis and treatment of this cancer is still a challenge. Here, we report a case in a 7-year-old boy diagnosed as having hepatic embryonal rhabdomyosarcoma and observed for 38-month since first diagnosis. To our knowledge, this patient has achieved the longest survival time for this cancer type with no recurrence or metastasis. This report describes the diagnosis and successful experience of comprehensive treatment for rhabdomyosarcoma to share with clinicians.

## Case Presentation

A 7-year-old boy was admitted to our hospital to examine an abdominal mass. He had no obvious clinical symptoms such as abdominal pain or weight loss. In addition, he had no significant personal or family medical history except that he was born premature. Physical examination revealed a mass (~9 × 8 cm) in the upper abdomen that was firm with poor mobility and no obvious tenderness. Laboratory test results showed normal levels of white blood cells, hemoglobin, platelets, and transaminase. However, the following levels were elevated: alkaline phosphatase, 244.1 U/L (range: 45–125 U/L); lactase dehydrogenase, 482 U/L (range: 120–250 U/L); C-reactive protein, 34.4 mg/L (range: 0–3.5 mg/L). In addition, the virus markers of hepatitis B and C were negative, and serum tumor markers showed normal levels of alpha-fetoprotein, carcinoembryonic antigen, and CA19-9. However, neuron-specific enolase was 30.45 ng/mL (range: 0–16.3 ng/mL), and CA72-4 was 7.94 U/mL (range: 0–6.9 U/mL). The rest of the laboratory tests were normal.

**Figure 1:**
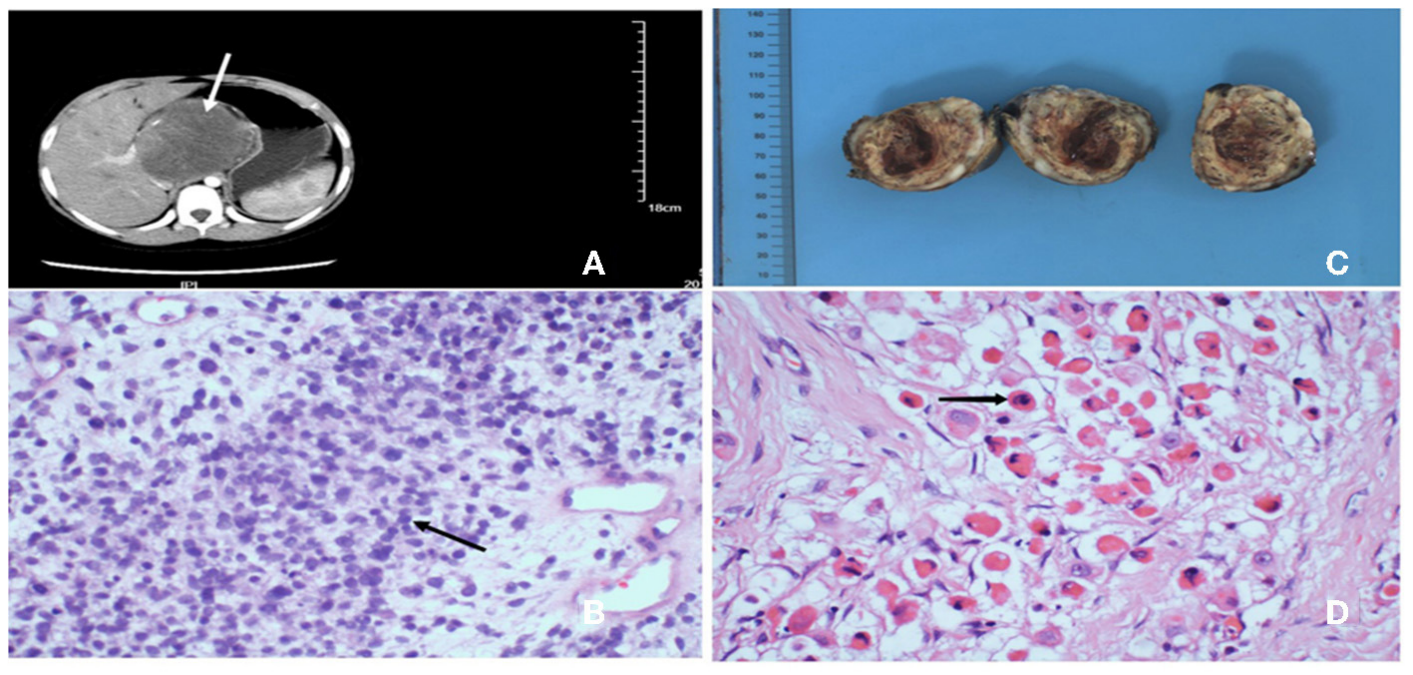
**(A)** Abdominal enhanced computed tomography showing a 7.5*7.3*7.7 cm high-density mass lesion involving left hepatic lobe, tending to be malignant. **(B)** After percutaneous liver biopsy, H&E stained showed the lession was composed of numerous large and small round cells, haphazardly oriented, containing variable amount of cytoplasm and prominent nucleolus (40×). **(C)** Postoperative specimen revealed a 5.5*5.0*4.0 cm well-defined soft mass with a gray/yellowish/reddish brown cut surface, in which hemorrhage, necrosis and cystic lesion could be seen. **(D)** H&E stained of the tumor revealed typical rhabdomyoblasts (black arrow) with oval nucleus, median nucleolus and eosinophilic cytoplasm (40×).

An ultrasound revealed a solid mass (83.3 × 79.4 × 106 mm) in the front of the upper abdomen spine, which is located behind the lesser curvature of the stomach, and the boundary with the left lobe of the liver was still clear. Abdomen contrast-enhanced computed tomography scans showed a mass-like high-density shadow (7.5 × 7.3 × 7.7 cm) in the left lobe of the liver, next to the lesser curvature of the stomach (Figure 1A). Subsequently, the positron emission tomography scan revealed a high metabolic mass between the liver and the stomach that was considered to be malignant, and the boundary with the liver and pancreas was not clear. Based on the tumor marker and imaging results, the patient was advised to undergo liver biopsy for definitive diagnosis.

He underwent percutaneous liver biopsy, and histopathological examination of the tumor cells displayed large and small round cells, haphazardly oriented, containing variable amount of cytoplasm and prominent nucleolus (Figure 1B). Immunohistochemical expression showed positive for vimentin and negative for chromogranin A, leukocyte common antigen, synaptophysin, and Wilm's tumor gene 1. Thus, the morphological and immunohistochemical features of the lesions accorded with clear cell sarcoma.

**Figure 2:**
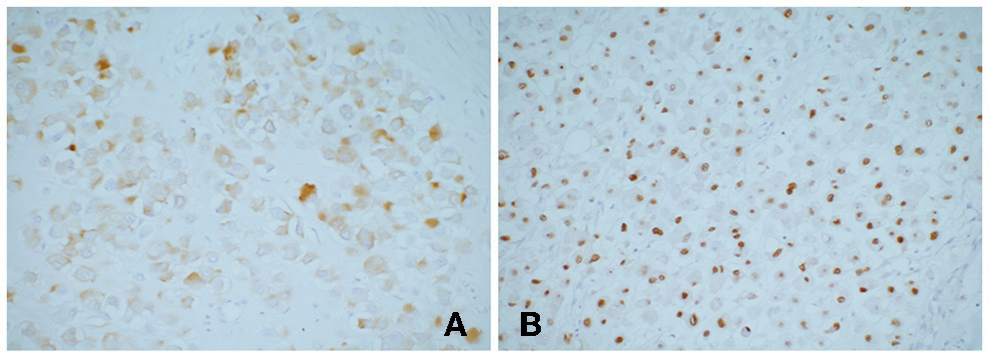
Immunohistochemical stains showing tumor cells **(A)** desmin positive; **(B)** myogenin positive. Myogenin is highly specific and sensitive for rhabdomyosarcoma, which currently used as the standard antibody for diagnosis.

The patient received two cycles neoadjuvant chemotherapy: 1.55 mg vincristine (given on days 1, 8, and 15 of a 21-day cycle), 1,500 mg ifosfamide (given on days 1–5 in a 21-day cycle), 100 mg etoposide (given on days 1–3 in a 21-day cycle), and responded well, including decreased tumor volume, so he was able to undergo surgery. Intraoperatively, a tumor was confirmed occupying the caudate lobe of the liver, rather than the left lobe of the liver as the abdominal computed tomography scan showed, and no infiltration of the surrounding organs and the cutting edge was identified. The gross specimen revealed a well-defined tenacious texture mass (5.5 × 5.0 × 4.0 cm; Figure 1C). Postoperative histopathological evaluation demonstrated typical rhabdomyoblasts with oval nuclei, median nucleoli, and eosinophilic cytoplasm (Figure 1D). Immunohistochemical analysis (Figure 2) was positive for vimentin, desmin, and myogenin and focally positive for smooth muscle actin. Finally, the diagnosis was established to be hepatic embryonal rhabdomyosarcoma based on typical histopathological features and diagnostic immunohistochemical staining.

After the patient had completely recovered from the surgery (11 days after surgery), the following postsurgical chemotherapy regimen was initiated: 1.55 mg vincristine (given on days 1, 8, and 15 of a 21-day cycle), 1,800 mg ifosfamide (given on days 1–5 in a 21-day cycle), and 100 mg etoposide (given on days 1–5 in a 21-day cycle). Chemotherapy was repeated every 3 weeks, and this therapy was well-tolerated without severe side effects. After three cycles, prophylactic radiotherapy in the liver tumor bed was started to obtain long-term tumor-free survival, and the total radiation therapy dose was 5040 Gy. After this therapy, he received another 10 cycles of chemotherapy: the first three cycles matched the previous postsurgical regimen; from the fourth cycle and on, the regimen changed to 1.55 mg vincristine (given on days 1, 8, and 15 of a 21-day cycle), cyclophosphamide 1,300 mg (given only on day 1), and 475 μg actinomycin D (given on days 1–5 in a 21-day cycle).To conclude, the patient was totally recievied 15 cycles of chemotherapy, including two cycles neoadjuvant chemotherapy and 13 cycles postsurgical chemotherapy. The overall treatment time which include surgical treatment, radiotherapy and chemotherapy was nearly 15 months.

**Figure 3:**
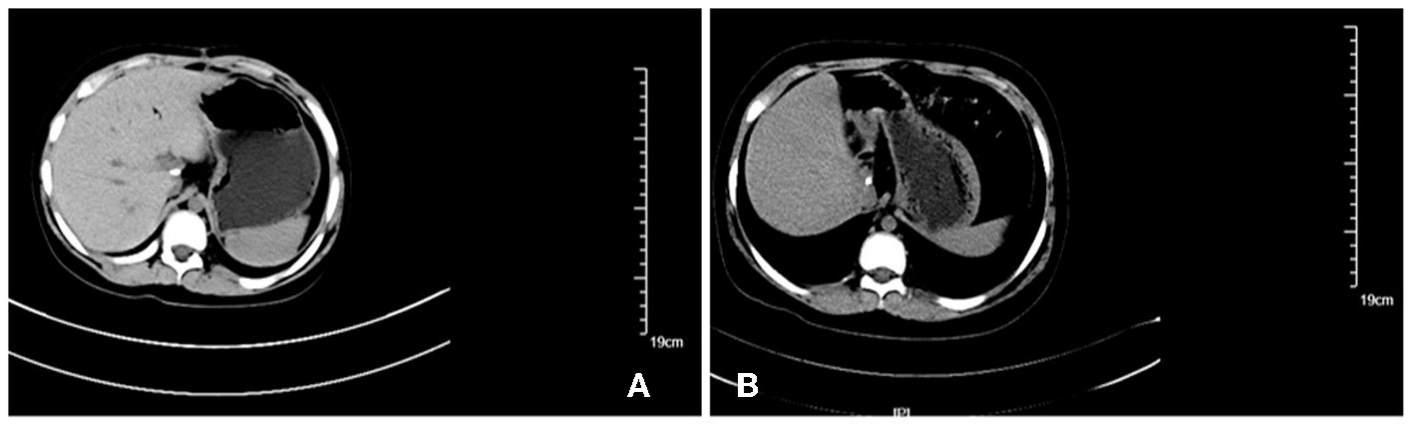
Imaging findings during follow up: there was no abnormal density shadow in the liver, which was consistent with the changes of hepatic caudate lobe resection. **(A)** abdominal computed tomography of the abdomen 2 months after the surgery; **(B)** Enhanced computed tomography of the abdomen 14 months after the surgery.

Although it was difficult to avoid infection or varying degrees of bone marrow suppression during chemotherapy, no serious life-threatening side effects occurred, and the life quality was guaranteed. Regarding the prognosis, the patient survives for 38 months since first being accepted to the hospital, and to date, no recurrence or metastasis has been found by abdominal computed tomography or abdominal ultrasound after treatment (Figure 3).

## Discussion

Primary rhabdomyosarcoma of the liver is extremely rare. Supplementary Table 1 summarizes the 20 cases of hepatic rhabdomyosarcoma reported (2–14) since 1956.

According to these reports, 11 cases presented clinical onsets with varying degrees of epigastric pain; three, with fever; two, with abdominal distension, two, with fatigue; and two, with shortness of breath. Imaging usually showed lesions in the right liver lobe. In addition, serological test results often indicated levels only slightly outside the normal range, with an occasional abnormal tumor marker. However, our patient had no clinical symptoms, and imaging revealed a mass on the caudate lobe of the liver, with an unclear boundary between the liver and pancreas. In addition, serum tumor markers showed abnormal levels of neuron-specific enolase and CA72-4. No reports to date have described the typical clinical symptoms and imaging findings of rhabdomyosarcoma, so the pathologic findings are regarded as the gold standard for diagnosis. For prognosis, the mean survival time of patients with hepatic rhabdomyosarcoma was 9.25 months (time span of 2–31 months) since initial diagnosis or symptoms. Notably, our patient was the only one alive for sure and had the longest disease-free survival by comprehensive treatment.

In a comparison analysis among reported previously cases, we calculated a misdiagnosis rate of 40% for patients with hepatic rhabdomyosarcoma. There are three main reasons for misdiagnosis of these patients. First, clinical manifestation can be highly variable which lack of typical clinical representations. Most symptoms depend on the location of the primary tumor, the presence or absence of metastases, and whether any complications developed. Second, the liver as a rare location of rhabdomyosarcoma has an insidious onset, make it easy to misdiagnose. Third, imaging findings of rhabdomyosarcoma have little diagnostic significance. However, they can provide useful assessment of the primary tumor, its size, and local and distant spread (15) and can be pivotal for the long-term follow-up of patients and early detection of recurrence.

Quite specially, our case is the only one misdiagnosed by a pathologic diagnosis rather than imaging findings or clinical symptoms despite the liver histopathological examination was seen as a gold standard in diagnosis of RMS. The reason might be pathological tissue collected by needle biopsy was too limited or contained too much necrosis material and it is hard for pathologists to effectively view the lesions and select the targeted protein for immunohistochemical staining. This episode highlights an important lesson: for disease diagnosis, we strongly suggest incisional biopsy rather than needle biopsy to ensure the retrieval of complete tissue for pathological diagnosis (16). If needle biopsy is chosen, it is better to use a larger diameter puncture needle or suggest the patient undergo repeat puncture biopsy to obtain enough pathological tissue for accurate diagnosis. That is to say, pathological diagnosis as the qualitative diagnostic evidence cannot be 100% correct.

Regarding treatment and prognosis, our patient was the only one who received the most comprehensive treatment, had the longest survival since diagnosis, and achieved a favorable curative effect. As Malempati and Hawkin (17, 18) suggest, most patients are stratified into high-, intermediate- and low-risk groups based on tumor size, invasiveness, nodal status, primary tumor site, and pathological and molecular parameters (PAX-fusion status) for tailored treatment. In addition, the overall cure rates have improved significantly thanks to the multimodal therapies, including surgery, radiation therapy, and chemotherapeutics (with various combinations of vincristine, actinomycin D, cyclophosphamide, etoposide, irinotecan, or ifosfamide). We closely studied recent cases of different locations (paratesticular, prostate, biliary and uterine) of RMS and found surgical therapy is the key basis and significant treatment for a better prognosis of patients with RMS except with orbital rhabdomyosarcoma (19–24). These available findings are consistent with our review. Of all 20 hepatic rhabdomyosarcoma cases reported, 16 patients for whom we know the treatment. Our case summary indicates that the mean survival time of patients who underwent surgery (10 patients) is 16.7 months, whereas of patients with non-surgical therapy (6 patients) is only 9.5 months. All patients who underwent surgical therapy received anatomic lobectomies (left/right hemihepatectomy or extended hemihepatectomy) based on the size and the site of the tumor, and there was no significant difference in surgical methods. It's important to note that our patient had a caudal lobectomy due to the site of the tumor, which is different from other patients that have been reported. Further research is needed to determine whether the location of the tumor affects prognosis of hepatic primary rhabdomyosarcoma.

**Figure 4:**
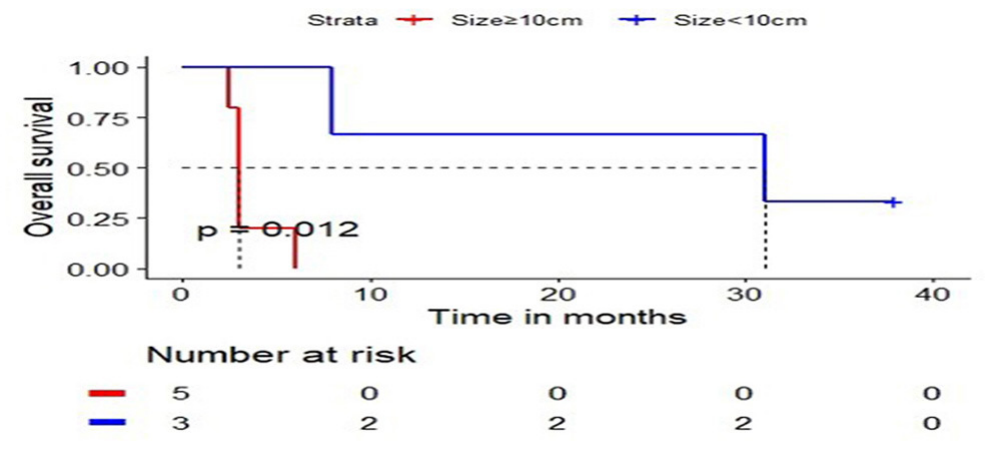
The relationship curve between tumor size and overall survival.

Among the cases reported, we grouped the cases by the diameter of tumor, which set 10 cm as a critical value. A total of eight cases were reported with explicit tumor size and survival state, including five cases with the largest tumor diameter of 10 cm or larger and three cases with a tumor diameter of <10 cm. We calculated and compared the overall survival time (Figure 4) for each case. From these results, we conclude that tumor size is negatively correlated with overall survival, indicating patients with larger hepatic rhabdomyosarcomas had shorter survival times.

In addition, we analyzed the cases according to different treatment regimens. The results indicated that despite the value of surgical care and the sensitivity of rhabdomyosarcoma to chemotherapy and radiotherapy, a single therapeutic strategy often fails to achieve the expected result regardless of the size of the tumor. Multidisciplinary comprehensive treatment can significantly prolong survival time (Supplementary Table 2). Among the reported cases, our patient has attained the longest survival time since initial diagnosis and was also the only one to receive neoadjuvant chemotherapy, surgery, and prophylactic radiotherapy and polychemotherapy. In contrast to chemotherapy for preventing any further spread or metastasis of the cancer, neoadjuvant chemotherapy aims to reduce the tumor size to allow for a complete resection at surgery. Another prognostic factor of survival is tumor histology. Patients with embryonal subtypes had the highest survival rates while those with pleomorphic and alveolar subtypes had the most aggressive cancer and worst prognosis (25). Furthermore, Malempati et al. (18) reported age as an independent prognostic factor in rhabdomyosarcoma; however, the study was limited by the number of cases and needs further evidence.

## Conclusion

Rhabdomyosarcoma, a malignant tumor with striated muscle differentiation, rarely occurs in liver. Here, we summarized all published hepatic rhabdomyosarcoma cases and reported a patient with the longest survival time for this cancer type with no recurrence or metastasis. This case report suggests incisional biopsy as the top preference in diagnosis, larger diameter puncture needle biopsy or repeat puncture biopsy is second preference. In terms of remedy, a comprehensive treatment based on surgery and chemotherapy have better effectiveness, and we strongly suggest that surgery should be carried out whenever resection is possible.

## Data Availability Statement

The original contributions presented in the study are included in the article/Supplementary Material, further inquiries can be directed to the corresponding author/s.

## Ethics Statement

Ethical review and approval was not required for the study of human participants in accordance with the local legislation and institutional requirements. Written informed consent was obtained from the patients' parents before clinical samples were collected. The patient and their parents were informed of the test results. Written informed consent was obtained from the patients' parents for the publication of any potentially identifiable images or data included in this article.

## Author Contributions

XuL and XiL developed the original idea, reviewed the literature, and wrote the manuscript. D-FY was the patient's doctor in charge and was responsible for collecting medical history. ML analyzed the histological characteristics of all pathologic sections. H-QX was responsible for collecting medical history and analyzing the statistical issues involved in this case. SZ was responsible for analyzing the imaging data. P-JG reviewed the literature and contributed to manuscript revision. All authors have read and approved the manuscript.

## Funding

This study was supported by the Science and Technology Development Program of Jilin Province; Grant Number: 20190103079JH and Youth Development Foundation of the First Hospital of Jilin University; Grant Number: JDYY102019004.

## Conflict of Interest

The authors declare that the research was conducted in the absence of any commercial or financial relationships that could be construed as a potential conflict of interest.

## Publisher's Note

All claims expressed in this article are solely those of the authors and do not necessarily represent those of their affiliated organizations, or those of the publisher, the editors and the reviewers. Any product that may be evaluated in this article, or claim that may be made by its manufacturer, is not guaranteed or endorsed by the publisher.
