# Supplementary material for: Case Report: Pediatric Hepatic Rhabdomyosarcoma With Maximum Lifetime
Source: Front Med (Lausanne). 2022 Apr 15;9:858219. doi: 10.3389/fmed.2022.858219 (PMC9051403; doi:10.3389/fmed.2022.858219)
Supplement: Supplementary file 1 [file Table_1.pdf]

Authors	Year	Age/Sex	symptoms	Extent of liver involvement: Imaging or autopsy characteristics	Initial diagnosis	Confirmed diagnosis (Histological classification)	Treatment	Outcome
Miller et al	1956	14/Female	Mild epigastric pain for 1 week, then developed severe right upper quadrant abdominal pain	This necrotic cystic mass measures 15.5×9.5×12 cm on the right lobe of the liver	N/A	Rhabdomyosarcoma	Heavy dose of x-ray therapy and radiation therapy then got total right hepatic lobectomy	N/A
Goldman et al	1969	65/Female	Steady pain in the right upper abdomen for 3 months, and the pain increased in severity and began radiating to the right lower chest,	Autopsy:the right lobe contained an ovoid tumor measured 35×15×10 cm	Hepatoma (based on clinical analysis)	Embryonal/ alveolar rhabdomyosarcoma with hepatocellular	Symptomatic treatment without any surgical procedure or chemotherapy	Died 3 months after the initial symptom
Babut et al	1976	N/A	N/A	N/A	N/A	N/A	N/A	N/A
Watanabe et al	1983	70/Male	Body weight loss for 5 months, non-productive cough and general fatigue for 3 months and slight fever for 2 months	Abdominal CT scan: non-capsulated multinodular liver tumors in the right lobe Autopsy: yellowish-brown multinodular tumors up to 5 cm in diameter in	hepatocellular carcinoma (based on liver	Pleomorphic rhabdomyosarcoma	Symptomatic treatment without any surgical procedure or chemotherapy	Died 8 months after the initial symptom
Shibata et al	1987	N/A	N/A	N/A	N/A	N/A	N/A	N/A
McArdle et al	1989	53/Male	Epigastric and midabdominal pain accompanied by anorexia,nausea and flatulence for 10 days	Abdominal CT scan: Large mass occupied the entire right lobe of the liver	Not clear, Fine-needle aspirate only revealed necrotic cellular debirs	Embryonal rhabdomyosarcoma	Surgical resection	Died 3 months from the initial symptoms
Bürrig et al	1994	69/Male	N/A	N/A	N/A	Pleomorphic rhabdomyosarcoma	N/A	N/A
Meyer-Pannwitt et al	1996	2 caese NA/Female	N/A	N/A	N/A	Pleomorphic rhabdomyosarcoma	The older woman: Hemihepatectomy The younger one: hemihepatectomy + adjuvant chemotherapy	Survived at least 9 or 15
Akasofu et al	1999	52/Male	General malaise and easy fatiguability for 2 months	Autopsy: 19×12×11 cm tumor occupied almost the entire right lobe; it was not encapsulated and had invaded the right adrenal gland, diaphragm, bilateral hepatic ducts, and inferior vena cava	Hepatocellular carcinoma (based on magnetic resonance imaging of the abdomen and CT angiography)	Phabdomyosarcoma	Symptomatic treatment without any surgical procedure or chemotherapy	Died 2.5 months from the initial symptoms
Huang et al	2003	8/Male	Abdominal pain, spiking fever and a rapidly growing abdominal mass for 1 week	Preoperative imaging : a large solid tumor in the right lobe of the liver	N/A	Pleomorphic rhabdomyosarcoma	Surgical resection (Extended right hepatectomy)	Died 2 months after the surgey because of the tumor recurrence with massive internal hemorrhage
Tutar et al	2007	6/Male	Pain in the right upper abdominal quadrant for 1 month	Dynamic CT imaging: 8 cm×6 cm cystic mass in the medial portion of the left liver lobe	Abscess or hydatid cyst of the liver (based on CT and	Embryonal botryoid-type rhabdomyosarcoma	Surgical resection (Extensive left hepatectomy)	N/A
Schoofs et al	2011	59/Female	Progressive upper abdominal discomfort and heartburn for 2 weeks	Magnetic resonance showed a lesion diameter 6 cm in segment 3 of the left liver lobe, which had stomach and pancreas invasion	Primary malignant hepatic tumor with intrahepatic metastasis (based on Magnetic resonance and CT)	Alveolar rhabdomyosarcoma	Surgical resection (Left hemihepatectomy) + Chemotherapy with doxorubicin (25 mg/m²) and ifosfamide(3g/m²) + Radiotherapy((5*4 gray)	Initial good response to chemotherapy and stable disease at 12 months after diagnosis; died 31 months after the first symptoms
Aassab et al	2012	25/Male	Pain in the right upper abdominal quadrant.	CT showed a large hypodense lesion in the right lobe of the liver measuring 136 mm.	N/A	Embryonal botryoid-type rhabdomyosarcoma	Polychemotherapy regimen with doxorubicin (50 mg/m²) ifosfamide (25 g/m²) and vincristine (14 mg/m²)	Died 3 months from the initial symptoms
Haider et al	2013	17/Male	N/A	N/A	N/A	Embryonal rhabdomyosarcoma	Multiple chemotherapies (doxorubicin, ifosfamide, dacarbazine; gemcitabine, paclitaxel; vincristine, actinomycin D, cyclophosphamide)	Died 31 months from the initial symptoms
Arora et al	2016	67/Male	Abdominal pain,abdominal distention and respiratory difficulty for 1 month.	Contrast-enhanced CT: a 14.5×12.3×9.1 cm lesion in the left hepatic lobe,which was seen bulging into the left subhepatic space, indenting over the stomach, compressing the pancreas and gall bladder	N/A	Embryonal rhabdomyosarcoma	Surgical resection (left hepatic lobectomy) + Chemotherapy with doxorubicin, ifosfamide and vincristine	Free from local recurrence and distant metastasis at 24 months of follow-up
Li et al	2017	40/Male	Without any symptoms	Macroscopically,a 4.5x4x4cm mass was poorly circumscribed and soft in the central region of left lateral lobe of the liver with apparently focal necrosis.	N/A	Embryonal rhabdomyosarcoma	Surgical resection (hepatic left lateral lobectomy)	No sign of recurrence or metastasis over 32 months of follow-up
Yin et al	2018	66/Female	A 5-year history of an abdominal mass without any symptoms but aggravated epigastric pain for the past 10 days	Operative biopsy: A large mass measuring about 20 ×15 cm in the right lobe of the liver, which adhered tightly to the omentum, mesentery, and intestines anteriorly, and to the abdominal wall and diaphragm posteriorly.	Hepatic cyst with rupture, hemorrhage, and infection (based on radiology findings and history of surgeries)	Pleomorphic rhabdomyosarcoma	Emergency laparotomy for hemostasis and right hepatic lobectomy without adjuvant chemotherapy	Died 3 months from surgery for malnutrition and multiple organ failure
Akki et al	2019	57/Female	Shortness of breath, epigastric discomfort and fatigue for 6	Magnetic resonance:a large(>18.0 cm) exophytic left hepatic lobe mass.	Intrahepatic cholangiocarcinoma with peritoneal carcinomatosis. (based on Imaging findings)	Spindle cell/sclerosing rhabdomyosarcoma	Surgical resection (partial hepatectomy, left hemi-diaphragm resection and omentectomy) +Chemotherapy with vincristine, cyclophosphamide, doxorubicin, and ifosfamide with etoposide	Disease-free for 12 months after adjuvant chemotherapy
Okazaki et al	2020	73/Female	Fever and right upper abdominal pain for 2 months	Contrast-enhanced CT:a 12×10cm lesion involving right hepatic lobe containing cystic and solid components.	RMS cell-like component (based on percutaneous liver biopsy)	Pleomorphic rhabdomyosarcoma	Surgical resection (right hepatic lobectomy) + Chemotherapy with trabectedin	Died 6 months from surgery since the rapid progression of the tumor
Present case	2020	7/Male	Investigation of a abdominal mass accidentally, without any clinical symptoms	Contrast-enhanced CT : a 7.5x7.3x7.7cm mass-like high-density shadow in the left lobe of the liver, next to the lesser curvature of the stomach	Clear cell sarcoma of liver (baesd on percutaneous trucut	Embryonal rhabdomyosarcoma	Neoadjuvant chemotherapy with vincristine , Ifosfamide, etoposide +Esection of caudate lobe tumor of liver + Polychemotherapy regimen with vincristine , Ifosfamide, etoposide and Actinomycin D +Prophylactic radiotherapy (5040cGy/28F)	So far no recurrence or metastasis was found of 38 months follow-ups since initial diagnosis, and we think he was clinically cured.
