# Supplementary material for: Case Report: Pediatric Hepatic Rhabdomyosarcoma With Maximum Lifetime
Source: Front Med (Lausanne). 2022 Apr 15;9:858219. doi: 10.3389/fmed.2022.858219 (PMC9051403; doi:10.3389/fmed.2022.858219)
Supplement: Supplementary file 2 [file Table_2.pdf]

Therapeutic regimen		tumor size	Outcome
chemotherapy alone	2012 25/Male	≥10cm	Died 3 months from the initial symptoms
	2013 17/Male	N/A	Died 31 months from the initial symptoms
surgery alone	1989 53/Male	N/A	Died 3 months from the initial symptoms
	2003 8/Male	N/A	Died 2 months after the surgey
	2007 6/Male	＜10cm	N/A
	2017 40/Male	＜10cm	No sign of recurrence or metastasis over 32 months of follow-up
	2018 66/Female	⩾10cm	Died 3 months from surgery
surgery combine chemotherapy	2016 67/Male	⩾10cm	Free from local recurrence and distant metastasis at 24 months of follow-up
	2019 57/Female	⩾10cm	Disease-free for 12 months after adjuvant chemotherapy
	2020 73/Female	⩾10cm	Died 6 months from surgery since the rapid progression of the tumor
surgery combine chemotherapy and radiationtherapy	2011 59/Female	＜10cm	Died 31 months after the first symptoms
	2020 7/Male	＜10cm	So far no recurrence or metastasis was found of 38 months follow-up, and we think he was clinically cured.
